# Editorial: Small Non-coding RNAs in *Streptococci*

**DOI:** 10.3389/fgene.2016.00192

**Published:** 2016-11-02

**Authors:** Mohamed A. Zorgani, Emilie Camiade, Roland Quentin, Marie-Frédérique Lartigue

**Affiliations:** ^1^ISP, Institut National De La Recherche Agronomique, Université Tours, Equipe Bactéries et Risque Materno-Foetal, UMR 1282Tours, France; ^2^Centre Hospitalier Régional Universitaire de Tours, Service de Bactériologie Virologie et Hygiène HospitalièreTours, France

**Keywords:** small RNAs, *Streptococcus*, *cis*-acting RNAs, *trans*-acting RNAs, CRISPR RNAs

Bacterial small RNAs (sRNAs) are post-transcriptional regulators of gene expression and the mechanisms by which this can occur have begun to be understood (Gottesman and Storz, 2011). In pathogenic bacteria, the importance of sRNAs-mediated regulation depends on a fine-tuning of the expression of various virulence genes (Papenfort and Vogel, 2010). For instance, RNAIII, a regulatory RNA from the pathogenic bacterium *Staphylococcus aureus*, controls the expression of different virulence factors such as *rot* (repressor of toxins), *spa* (surface protein A), *sa1000* (fibrinogen binding protein), or *coa* (coagulase) (Boisset et al., 2007). Considering the functional roles of sRNAs, they are categorized in two major classes: (*i*) *cis-*acting elements located/acting on untranslated regions (UTRs) of a translated mRNA which controls the expression of their enclosed gene(s) through the modulation of their secondary structures or stability; and (*ii*) the *trans*-acting sRNAs that comprise the *cis*-encoded sRNAs, known as antisense RNAs (asRNAs), and the *trans*-encoded sRNAs which generally present imperfect base-pairing with the mRNA targets (Zorgani et al., 2016).

The goal of this special research topic is to bring together research reviews and original articles on sRNAs, their identification and the characterization of their mode of action in *Streptococci*. Streptococcal species can colonize and invade humans and animals. They mostly exhibit an asymptomatic interaction within their hosts. However, several of them, such as *Streptococcus pyogenes* and *Streptococcus pneumoniae*, are well-known pathogenic species responsible for severe and live-threatening infections in humans (Lamagni et al., 2009). The development of biocomputational and high throughput screening methods, like next generation sequencing, led to the discovery of hundreds of sRNAs encoded by the bacterial genome, which represent 3–5% of total number of annotated genes regardless of the studied species (Siezen et al., 2010). Currently, the main challenges in the study of these molecules in *Streptococci* are their identification, as well as the identification of their potential targets. Three review papers published in this research topic reported that a large number of putative regulatory sRNAs were recently identified in different streptococcal species (Cho and Kim; Patenge et al.; Wilton et al.).

Most of these studies focus on three pathogenic species: *S*. *pyogenes* and *S. pneumoniae* and the opportunistic pathogen involved in neonatal infections, *Streptococcus agalactiae*. At present, 75 sRNAs have been identified in *S. pyogenes* (Perez et al., 2009; Patenge et al., 2012; Tesorero et al., 2013) and 179 sRNAs in *S. pneumoniae* (Kumar et al., 2010; Tsui et al., 2010; Acebo et al., 2012; Mann et al., 2012). In *S. agalactiae*, 197 sRNAs were predicted *in silico* and 10 of them were validated by northern blot (Pichon et al., 2012). More recently, 125 sRNAs were identified by dRNA-seq (differential RNA-sequencing) in the strain *S. agalactiae* NEM316 (Rosinski-Chupin et al., 2015).

Although, several sRNAs have been identified to date in *Streptococci*, very few are characterized. For instance, in *S. pyogenes*, only three sRNAs were characterized: (i) FasX, which binds to the 5′ end of *ska* mRNA and activates its translation by avoiding degradation with RNase J1 (Ramirez-Peña et al., 2010); (ii) RivX, which affects the expression of *emm, scpA, mga*, and *speA* mRNAs (Roberts and Scott, 2007); and (iii) Pel, a pleiotropic sRNA which affects the expression of different virulence factors (*emm, sic*, and *speB*) and encodes at the same time an hemolysin (Mangold et al., 2004).

In *S. pneumoniae*, a combination of biocomputational approaches, transcriptomic analysis and RNA-seq were used to identify sRNAs encoded by *S. pneumoniae*. While an *in silico* study identified 128 sRNAs located in intergenic regions (Livny et al., 2008), 50 sRNAs were identified by microarray analysis (Kumar et al., 2010), and 88 sRNAs by RNA-seq (Acebo et al., 2012). A recent study published in our special research topic demonstrated that csRNAs (*cia*-dependent small RNAs) are involved in competence control in *S. pneumoniae* R6 (Laux et al.). The csRNAs are non-coding *trans*-acting sRNAs transcribed from the promoter associated to CiaRH two-component system. They generally present a complementarity to the Shine-Dalgarno sequence and the translation initiation codon (AUG). The authors showed, by using reporter gene fusions, that deletion of a single or two csRNAs was not sufficient to impair competence gene expression. In contrast, combination of three csRNAs, or mutations in the competence gene *comC* and its complemented csRNAs, completely blocked *S. pneumoniae* competence. This study demonstrated that csRNAs are important players in competence regulation for *S. pneumoniae*.

Two original research articles focused on sRNAs in *S. agalactiae* were included in the present topic. The first study is about RNAII, an asRNA involved in replication control and encoded by pMV158 plasmid (López-Aguilar et al.). One of the main finding is that RNAII do not requires the formation of a kissing complex for efficient binding to its mRNA target or for inhibition of *repB* expression. The *repB* gene encodes the replication initiator protein of the pMV158 plasmid. However, site-directed mutagenesis on the 5′ and 3′ regions on the 5′-tail of RNAII, revealed that the entire 5′-tail on the asRNA is mandatory for efficient *repB* translation *in vitro* and pMV158 replication *in vivo*.

The second study described the distribution of the type II-A CRISPR-Cas system among the different *S. agalactiae* genetic lineages (Lier et al.). CRISPR RNAs (Clustered Regularly Interspaced Short Palindromic Repeats RNAs) are sRNAs that target invading cognate nucleic acids (Chylinski et al., 2013). Beside the ubiquitous presence and the high sequence polymorphism of CRISPR1 spacers, one of the main finding of this study is that strains belonging to the “highly virulent” sequence type 17 clone showed a lower number of spacers. This result suggests that sequencing of the CRISPR1 locus can be used as an alternative tool for phylogenetic studies.

The sRNAs widespread in the bacterial kingdom, and particularly in S*treptococci*, highlights their importance in regard to regulatory networks and pathogenesis and open new perspectives for future therapeutic and industrial applications. This special research topic was the opportunity to review the different sRNAs categories in *Streptococci* and to emphasize their role in gene regulation, physiology, and virulence.

## Author contributions

MZ: Wrote the manuscript; ML, EC, and RQ: Participated in manuscript correction.

### Conflict of interest statement

The authors declare that the research was conducted in the absence of any commercial or financial relationships that could be construed as a potential conflict of interest.
